# Physician and nurse supply in Serbia using time-series data

**DOI:** 10.1186/1478-4491-11-27

**Published:** 2013-06-17

**Authors:** Milena Santric-Milicevic, Vladimir Vasic, Jelena Marinkovic

**Affiliations:** 1 Institute of Social Medicine & Center School of Public Health and Health Management, Faculty of Medicine, University of Belgrade, Dr Subotica 15, 11000, Belgrade Serbia; 2 Department of Statistics and Mathematics, Faculty of Economics, University of Belgrade, Belgrade, Serbia; 3 Institute of Statistics and Informatics, Faculty of Medicine, University of Belgrade, Serbia Dr Subotica 15, 11000, Belgrade, Serbia

## Abstract

**Background:**

Unemployment among health professionals in Serbia has risen in the recent past and continues to increase. This highlights the need to understand how to change policies to meet real and projected needs. This study identified variables that were significantly related to physician and nurse employment rates in the public healthcare sector in Serbia from 1961 to 2008 and used these to develop parameters to model physician and nurse supply in the public healthcare sector through to 2015.

**Methods:**

The relationships among six variables used for planning physician and nurse employment in public healthcare sector in Serbia were identified for two periods: 1961 to 1982 and 1983 to 2008. Those variables included: the annual total national population; gross domestic product adjusted to 1994 prices; inpatient care discharges; outpatient care visits; students enrolled in the first year of medical studies at public universities; and the annual number of graduated physicians. Based on historic trends, physician supply and nurse supply in the public healthcare sector by 2015 (with corresponding 95% confidence level) have been modeled using Autoregressive Integrated Moving Average (ARIMA) / Transfer function (TF) models.

**Results:**

The ARIMA/TF modeling yielded stable and significant forecasts of physician supply (stationary R^2^ squared = 0.71) and nurse supply (stationary R^2^ squared = 0.92) in the public healthcare sector in Serbia through to 2015. The most significant predictors for physician employment were the population and GDP. The supply of nursing staff was, in turn, related to the number of physicians. Physician and nurse rates per 100,000 population increased by 13%. The model predicts a seven-year mismatch between the supply of graduates and vacancies in the public healthcare sector is forecasted at 8,698 physicians - a net surplus.

**Conclusion:**

The ARIMA model can be used to project trends, especially those that identify significant mismatches between forecasted supply of physicians and vacancies and can be used to guide decision-making for enrollment planning for the medical schools in Serbia. Serbia needs an inter-sectoral strategy for HRH development that is more coherent with healthcare objectives and more accountable in terms of professional mobility.

## Background

Policy makers often promote strategic planning for human resources for health (HRH) as a part of strategies aimed at improving healthcare system performance [1]. Strategic HRH planning for attaining improved health goals and objectives depends on understanding the interplay among many factors within and beyond the healthcare system. Those factors include: economic policies, legislation, rules and procedures that guide health workforce production, education, deployment, performance, payment and management, as well as structures, programs, and action plans designed to be operated and delivered by a range of providers in settings with different socio-economic and demographic characteristics; all of these factors responding to environmental threats and targeting a changing demographic structure [2-15]. Along with that, HRH planning can include projections that identify cross-cutting problems regarding HRH production, employment and management, such as the relative attractiveness of employment or practice in the health professions, the role of the private sector and migration of health professionals and the population [1-13].

During the last sixty years, various tools have been developed and used for HRH planning [2-12,14-16]. Developed countries often used workforce supply and demand methods based on population needs-based requirements, others have used benchmarking or a combination [2-12,14,15] There are qualitative approaches (for example, the Delphi method [11]), and some studies use quantitative dynamic modeling of HRH stock and flow [14] that may include sensitivity analyses [15]. This paper describes a trend analysis, using existing data to anticipate supply and demand issues in Serbia.

A country may lack a coherent plan for HRH development, as well as valid data describing HRH shortages or excesses because of inaccurate data, conflicts with general policy planning, the presence of a significant private sector, or lack of a responsible body and support system to promote planning [11].

In all former Yugoslav republics including Serbia, HRH planning was driven by simple, normatively-determined physician/dentist/nurse/pharmacists-to-population ratio. This was done in a diffuse manner with the Ministry of Education and the Ministry of Health sharing responsibility, but the former had no legal obligation to consult with the Ministry of Health on the number of medical students enrolled in medical school and could act independently in setting targets [16-19]. In the South East European countries, including Serbia, systematic and strategic workforce planning has been underdeveloped, and, important for this region in recent decades, an understanding of migratory flows is lacking [16]. For example, Romania has a binding *numerus clausus* but there remains a relatively stable number of vacancies in the public sector mainly due to limitations in teaching capacity that have not been adjusted upward. Overproduction and overspecialization in Bulgaria have been the result of the lack of clear criteria for HRH planning [16].

Specific studies of HRH planning are limited in Serbia [20-25]. Since the Second World War, the public sector has been the major employer and producer of health workforce in the Republic of Serbia. Before 1961, the country had successive five-year economic plans, which included HRH. Due to scarce teaching capacity, a *numerus clausus* was in place and regulated the number of graduates for that reason instead of population needs. The application of HRH decision-making was decentralized to ‘self managed interest communes’ that aimed to increase population access and equity, but these were not connected to the overall number of graduates [20,23].

From 1961 to 2010 there have been three formal HRH strategies: the first two were long-term strategies created within health care development plans, for 1990 [20] and 2000 [21] respectively; the third was part of planned activities for the reconstruction of the health care provider network during 2005 to 2010 [22].

The first HRH development plan for 1990 followed the new Constitution (in 1974), the Labor Law, and the Law on Education of 1976 that brought broader decentralization to all former Yugoslav republics and provinces [25]. However, after the global oil crises in 1976 the economy stagnated, national debt rose and separatist tensions emerged [25]. By 1978 the number of employed physicians and enrolled medical students slowly increased, but in 1979 the number of enrolled medical students doubled and stayed at that level for several consecutive years [24]. Financial restrictions on the healthcare system and the elimination of private practice forced many health workers to emigrate. At the beginning of the 1980s, Constitutional amendments limited the autonomy of republics and Serbian provinces and this led to a new approach using centralized HRH planning.

A new development plan for Serbia was enacted and was applied between 1982 and 2000 [21]. It included a reduction of enrollment in medical studies, increased vocational education and specification of the number of posts based on health worker to population ratio and it also sanctioned private practice for dentists in 1987 and for pharmacists and physicians in 1989. However, military conflicts and Yugoslavia’s break-up during the 1990s curtailed the inter-sectoral activity over HRH development. Private practitioners were not able to support themselves with fees and many health professionals emigrated.

At the end of the 20th century, Serbia was an economically degraded and isolated country, overburdened with hyperinflation and experiencing an influx of refugees and internally displaced persons (both healthcare workers and patients). Many health development plans were subsequently proposed by various experts, but none were formally accepted.

In 2000, the new Ministry of Health collaborated with the World Bank on a master plan for the reconstruction of the healthcare provider network and a new HRH strategy for 2006 to 2010 [22,26]. In order to fit with a reconstructed network of public healthcare providers, and attain equity in access, and to increase efficiency, a recommended reduction in public sector staff was aligned with estimates of an increase in the private sector; the private sector was expected to absorb these workers or attract recently retired workers. Despite the fact that unemployment of medical workers was high, and the training of the health workforce at state faculties was publicly funded, as were health care costs, the enrollment and graduation rate for medical studies were not priority issues in the HRH strategy 2006 to 2010. The planning process in place did make use of several demand-based scenarios for primary, secondary and tertiary healthcare institutions and included population and economic growth, healthcare services utilization, and performance benchmarking for the public sector.

Subsequently, regulations on physician and nurse staffing and operational staffing norms were adopted [27,28]. The Law of Private Entrepreneurs (2005) and the Law of Private Companies (2004) allowed the establishment and operation of private medical and dental practices or companies with limited liability. The Health Care Law of 2005 described the scope of health care services private health care providers could provide.

Although staffing ratios had been set, there were no explicit boundaries regulating deviations from standards such as in Slovenia where a 10% variance was allowed [19]. In Serbia, there were variances of up to 2.74 times for physician density and over three-fold for specialists; up to 1.98-fold for nurse density and over six-fold for midwives (Table 1). This was primarily due to population and health workforce rural-to-urban migration, differences in natural population increase, and noncompliance with staffing rules with little flexibility for health workforce movement. Nevertheless, 95% of physicians and nurses had permanent full or part-time employment. Meanwhile, annual unemployment for physicians had been growing by 5.6% and by 1.5% for nurses from 2000. Two-thirds of approximately 2,000 unemployed physicians were aged less than 30 years, and half of almost 10,000 unemployed nurses were under 25 years of age [24].

**Table 1:** Districts with the highest and the lowest number of health workers in the public healthcare sector per 100,000 population in the Republic of Serbia

**Year**	**Districts with the highest number of health workforce (physical persons) per 100,000 population**				**Districts with the lowest number of health workforce (physical persons) per 100,000 population**			
	**Nisavski**		**Belgrade**		**Sremski**		**Macvanski**	
	**Physician**	**Nurse**	**Physician**	**Nurse**	**Physician**	**Nurse**	**Physician**	**Nurse**
2005	428	631	353	693	143	332	186	430
2006	437	634	355	712	145	324	189	430
2007	437	673	358	725	151	331	195	442
2008	443	700	368	745	162	343	205	461
2009	421	676	368	742	175	364	206	455
2010	444	700	371	748	178	373	206	465

Source: Institute of Public Health of Serbia, Health Statistical Yearbook of Republic pf Serbia1. Belgrade: Institute of Public Health of Serbia; 2012.

The new Health Development Plan for the 2010 to 2015 period [29] anticipated the development of a parallel HRH strategy but it has not yet been created. The existing Health Care Law was extended with new articles by which the Ministry of Health annually sets the highest number of posts for each public healthcare institution under their control. Current annual staffing targets and the minimum required number and skill-mix of workers were unchanged and remain based on population number per square kilometer, and the population age and sex structure. Performance measures for public healthcare institutions were also unaffected [27,28].

This study explores these past planning approaches (between 1961 and 2008), and uses trend data from those years to model physician and nurse supply to determine if a longer term projection model can be applied in Serbia. The study purpose is to meet the requirement for the inter-sectoral activity over HRH development set out in the new Health Development Plan between 2010 and 2015 period.

## Methods

### Study design and data sources

The method used to develop estimates of the Serbian physician and nurse workforce made use of a multivariate Autoregressive Integrated Moving Average (ARIMA) approach. The predicted values (Y-variables) were the estimated number of physicians and nurses. The analysis focused on the total number of physicians (y1: general practitioners and specialists) and nurses (y2: general, pediatric nurses and midwives with secondary and higher education) employed in the public healthcare sector of Serbia from 1961 to 2008 and their forecast numbers as of 2015. Other variables used in the study were the annual population (x1: estimates and census data for 1961, 1971, 1981, 1991 and 2002); GDP (x2: real value at 1994 prices); inpatient care discharges (x3: proxy to physicians’ and nurses’ productivity in secondary and tertiary healthcare institutions), outpatient care visits (x4: proxy to physicians’ and nurses’ productivity in primary healthcare institutions including prevention, curative and rehabilitative visits in ordination and at home); students enrolled in the first year of medical studies at state universities (x5: proxy of higher education enrollment policy); and graduated physicians (x6: a proxy for higher education production). The analyses were separated into two periods, 1961 to 1982, and 1983 to 2008 in order to explore the shift in policy predictors of HRH planning in Serbia.

Data on physicians, nurses, outpatient care visits and inpatient care discharges in the network plan of the health institution in the Republic of Serbia as of 2000 (the public healthcare sector) were obtained from the Institute of Public Health of Serbia [30]. The public healthcare system is the major employer of the health workforce in Serbia and is financed predominantly via compulsory health insurance taxes and public taxes. HRH data from the private practice sector and other institutions that were not included in the public healthcare sector were not available with precision and were not included in the study. A precise overview of the number of entrepreneurs, companies and personnel that provide health care services is not available from public sources; the Republic Statistical Office publishes aggregate data related to the activity of ‘health and social work’ but this is not precise enough to use. The methods of data collection for the private sector have significant limitations in the Serbian context but some data are instructive [31]. Recent estimates are that the private sector provides only 3% of the overall healthcare services in Serbia making the estimate presented here broadly applicable [31]. The Medical Chamber of Serbia has information on licensed and unlicensed and employed and unemployed physicians. Of the total of 29,847 licensed physicians, 3,618 were private practitioners (12.12%). Adding unlicensed physicians to the count, that proportion falls to 11.49%. The Medical Chamber reports 1,761 unemployed doctors, a little less than is registered in the National Employment Bureau database. The number of unemployed doctors equals 5.90% of all licensed physicians (or 5.59% of licensed and unlicensed physicians). In 2013, there were 1,668 entities in the private sector, with 155 having opened and subsequently closed. Private practitioners are employed at 11 general hospitals, 123 polyclinics, and 631 physicians’ offices.

Data on population size, GDP and enrolled and graduated students were taken from the Statistical Office of the Republic of Serbia [32]. The analysis of the educational sector captures data on all the state Medical Faculties’ students in Serbia (financed via public taxes) but does not include enrollments in postgraduate or specialist studies. At the time of the analysis, there were no graduation data from private faculties as they have only been recently established [32]; the estimated annual intake at private faculties is less than 3% of the total enrollment at state faculties [24]. For consistency reasons, all data refer to the Republic of Serbia and exclude data from Kosovo and Metohija.

### Statistical analyses

Basic descriptive statistics of variables in the study are given in Table 2. The table includes longitudinal analysis of the relationship between selected potential predictors and employment of physicians and nurses, included analyses of all variables time-series (from 1961 to 2008) and a trend for 2015 including corresponding 95% upper confidence level (UCL) and lower confidence level (LCL).

**Table 2:** Basic descriptive statistics of all variables in the study in the period 1961 to 2008

**Name**	**Labels**	**Maximum**	**Minimum**	**Net change**
y1	number of physicians	20668	4618	3.24
y2	number of nurses	42480	6422	4.10
x1	population size number of inhabitants	7897937	6678239	0.20
x2	GDP value in real prices	48857	13662	2.75
x3	number of inpatient care discharges (in thousands)	1214	379	2.51
x4	number of outpatient care visits (in thousands)	50261	21849	1.20
x5	number of students enrolled at the first year of medical studies (at state faculties)	3946	945	1.35
x6	number of graduated medical doctors (at state faculties)	1724	553	1.68

Predictors of physician and nurse employment have been included in time-series models for the period 1983 to 2008 (potential predictors: x1, x2, x3, x4, x5, and x6) to forecast the numbers of physicians and nurses (key dependent variables: y1 and y2) employed from 2009 to 2015. Forecast outputs include absolute numbers of physicians and nurses with a corresponding 95% LCL and UCL. These estimates represent the workforce employed in the Serbian public healthcare sector per year and assume the relationships among the observed input variables (number of population, GDP, outpatient visits, inpatient discharges, enrollments and graduates at state medical universities) will not change significantly by 2015. Because of the world economic crisis, whose effects started to influence the Serbian economy in 2009, we included a ‘pessimistic’ scenario of GDP contraction (that is, GDP 95% LCL) instead of the GDP central projections. Forecasted outputs will differ from their realized annual values by 2015 if identified predictors or the HRH planning approach significantly change during the period between 2011 and 2015.

The Autoregressive Integrated Moving Average (ARIMA)/Transfer function (TF) time-series models [33] were the statistical methods applied in this study. The ARIMA/TF procedure also includes an Expert Modeler component that identifies and estimates an appropriate model for each output variable series and predicts its values. The specification of the model also identifies outliers. The model’s stability, significance and fit have been tested with stationary-R^2^ and Ljung-Box statistics. The Kolmogorov-Smirnov Z test has been used to verify normal distribution of residuals in the model. The statistical package was IBM SPSS Statistics (version 20). Software is available from IBM SPSS Statistics 20 Information Center [34].

## Results

### Physician and nurse deployment in the public healthcare sector of Serbia from 1961 to 2008

From 1961 to 1982, the number of employed physicians increased by 174%; the number of nurses by 282%, the population by 15%, GDP by 200%, the number of inpatient discharges by 132%, the number of outpatient visits 67%, the number of enrollments in first year medical studies by 206%, and the number of graduated physicians by 114%. From 1961 to 1982, change in the employment of physicians was significantly related only to GDP change while nurse employment grew independently of changes in the independent variables. Both physician and nurse employment models were statistically stable without outliers and with a normal distribution of residuals (Table 3). The number of inpatient care discharges was related to the number of employed physicians and GDP, while the number of outpatient care visits was related only to the number of employed physicians.

**Table 3:** Transfer function models from the first period 1961 to 1982

**Dependent variable (labels and name)**	**Potential predictors in start model (name only)**	**Significant predictors in final model (name only)**	**Model type**	**Stationary R**^**2**^	**Number of outliers**	**Q-stat (*P *value)**	**Z-stat (*P *value)**
Physicians (y1)	x1, x2, x3, x4,x5, x6	x2	TF (0,1,0)	0.63	0	1.52 (0.96)	0.81 (0.52)
Nurses (y2)	y1, x1,x2, x3,x4, x5, x6	none	ARIMA (0,2,0)	0.76	1	4.67 (0.59)	0.93 (0.35)
Inpatient care discharges (x3)	y1, y2,x1, x2,x4, x5, x6	none	ARIMA (0,1,0)	0.96	3	8.30 (0.22)	0.89 (0.40)
Outpatient care visits (x4)	y1, y2,x1, x2,x3, x5, x6	y1	TF (0,1,0)	0.61	0	6.00 (0.42)	0.70 (0.72)
Students enrolled in the first year of studies (x5)	y1, y2,x1, x2,x3, x4, x5, x6	x6	TF (0,1,0)	0.98	4	0.60 (0.99)	0.55 (0.92)
Graduated medical doctors (x6)	y1, y2, x1, x2, x3, x4, x5, x6	none	ARIMA (0,1,0)	0.85	3	3.85 (0.70)	0.84 (0.48)

Legend: stationary R^2^ - measure of goodness of fit of model. Range is from negative infinity to 1; Q-stat - is Ljung-Box Q statistics that test the null hypotheses of no autocorrelation in residual series; Z-stat - is Kolmogorov-Smirnov statistics that test the null hypotheses of normal distribution of residual series.

In the second period, from 1983 to 2008, the numbers of employed physicians and nurses each increased by about 43%. The population decreased by 6%, GDP by 50% and the number of outpatient visits by 11%. However, the number of inpatient discharges increased by 28%. Both the number of students enrolled in first year medical studies and the number of graduated physicians decreased by 50%. Physician employment correlated with population and GDP, while nurses correlated to the number of employed physicians (Table 4).

**Table 4:** Transfer function models from the second period 1983 to 2008

**Dependent variable (labels and name)**	**Potential predictors in start model (name only)**	**Significant predictors in final model (name only)**	**Model type**	**Stationary R**^**2**^	**Number of outliers**	**Q-stat ( *P *-value)**	**Z-stat ( *P *-value)**
Physicians (y1)	x1, x2, x3, x4, x5, x6	x1, x2	TF (0,1,0)	0.71	0	5.35 (0.50)	0.63 (0.82)
Nurses (y2)	y1, x1, x2, x3, x4, x5, x6	y1	TF (0,1,0)	0.92	2	7.34 (0.29)	0.53 (0.94)
Inpatient care discharges (x3)	y1, y2, x1, x2, x4, x5, x6	x2	TF (0,1,0)	0.78	1	7.34 (0.29)	0.51 (0.96)
Outpatient care visits (x4)	y1, y2, x1, x2, x3, x5, x6	y1	TF (0,1,0)	0.44	0	6.31 (0.39)	0.59 (0.88)
Students enrolled in the first year of studies (x5)	y1, y2, x1, x2, x3, x4, x5, x6	none	ARIMA (0,1,0)	0.73	1	4.97 (0.55)	0.67 (0.77)
Graduated medical doctors (x6)	y1, y2, x1, x2, x3, x4, x5, x6	none	ARIMA (0,1,0)	0.23	1	4.73 (0.58)	0.68 (0.74)

Legend: stationary R^2^ - measure of goodness of fit of model. Range is from negative infinity to 1; Q-stat - is Ljung-Box Q(6) statistics that test the null hypotheses of no autocorrelation in residual series; Z-stat - is Kolmogorov-Smirnov statistics that test the null hypotheses of normal distribution of residual series.

The physician-to-nurse ratio remained the same at 1:2. However, the population, GDP, inpatient care discharges and outpatient care visits were not significantly related to the number of enrolled students in first year medical studies (x5) or graduated physicians (x6).

**Figure 1:**
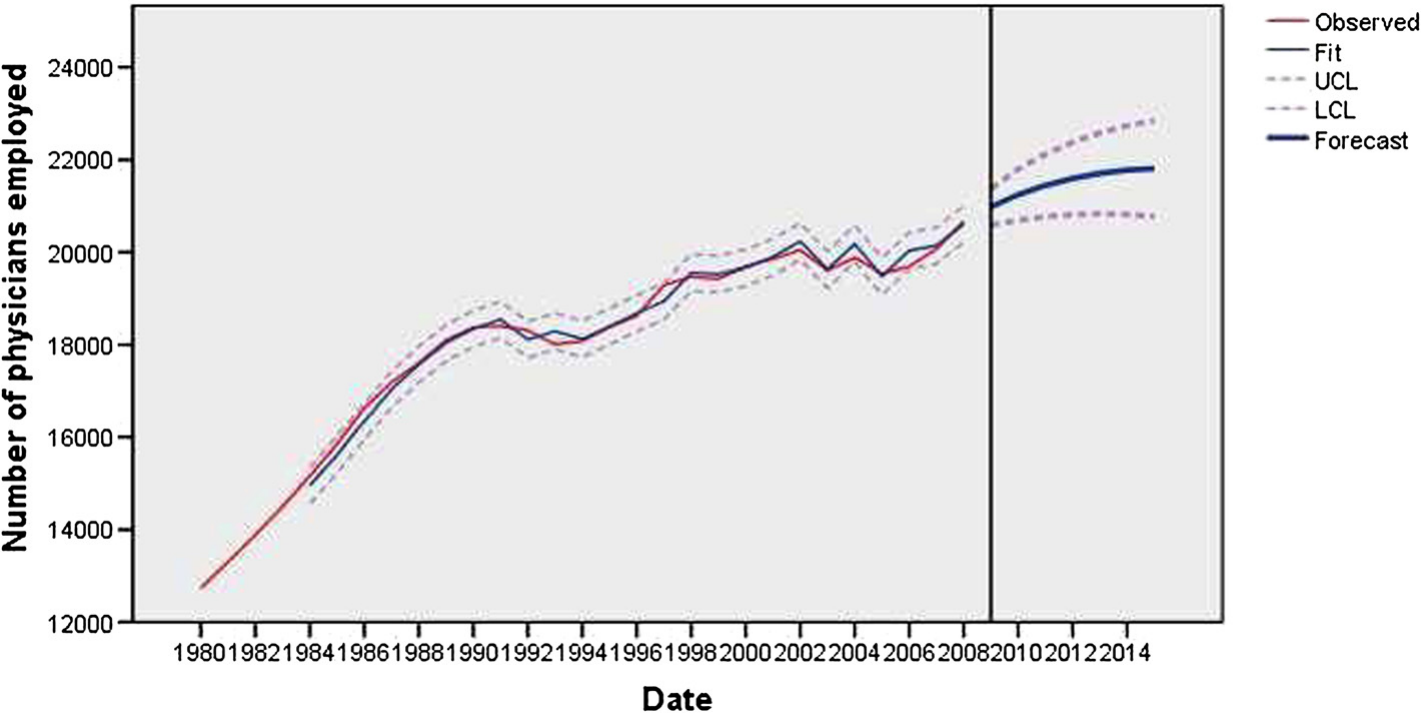
Observed and fitted number (with 95% LCL and UCL) of physicians employed in the public healthcare sector of Serbia (1983 to 2008) and the forecast by the year 2015.

**Figure 2:**
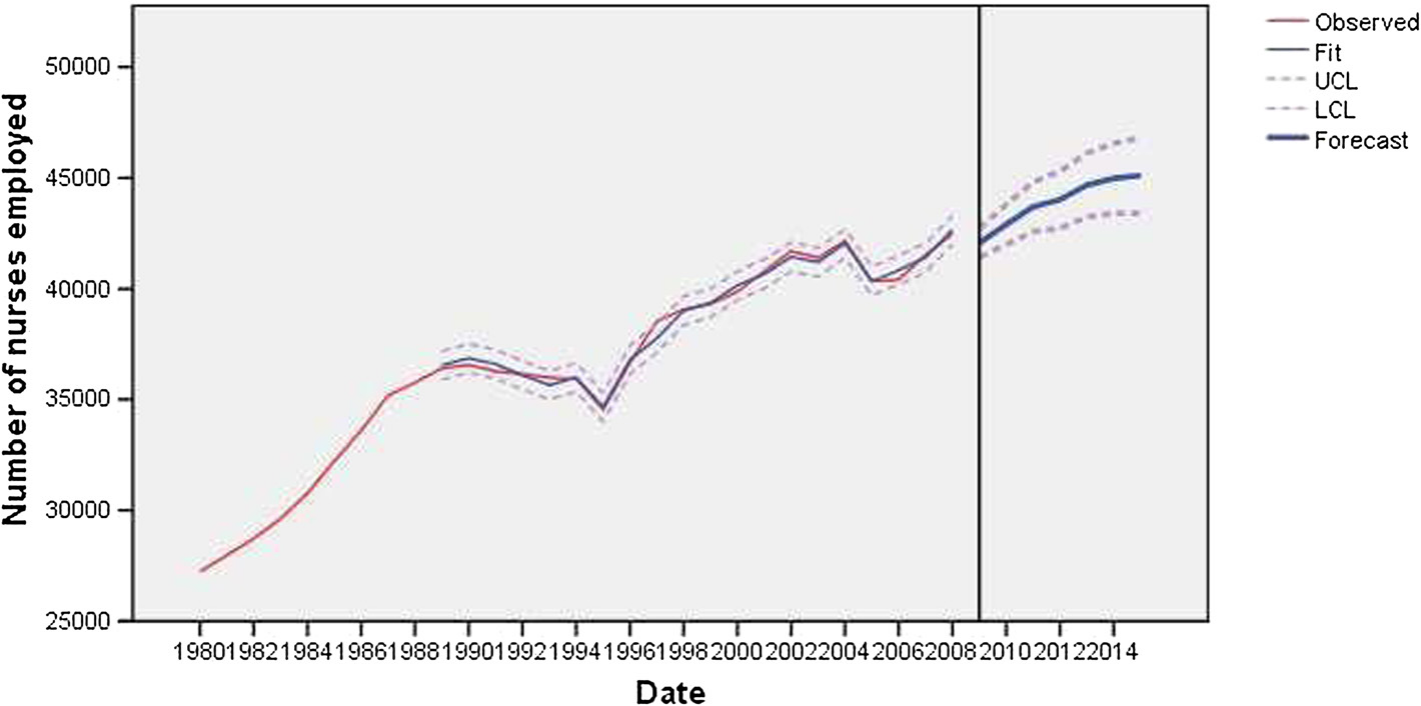
Observed and fitted number (with 95% LCL and UCL) of nurses employed in the public healthcare sector of Serbia (1983 to 2008) and the forecast by the year 2015.

The physician employment model was statistically stable, without outliers and with a normal distribution of residuals (Figure 1). The nurse employment model was statistically stable, with two outliers, and with a normal distribution of residuals (Figure 2). The modeled non-standard values in the nurses’ employment model were: additive in 1995 (*t* = −8.22; *P* < 0.01) and level shift in 2005 (*t* = −3.61; *P* < 0.01). In 1995, the nursing staff was significantly reduced, by about 3.88% due to flexible retirement and beneficial disability pensions, a decrease in deployment in 2005 by about 4.27% due to planned reductions in staffing and early retirement schemes in the public healthcare sector.

In the second period, ARIMA/TF models of almost all input variables had outliers, with the exception of the model of outpatient care visits.

The ARIMA (1, 2, 0) model of the population size time-series had four outliers: these were characterized by a level shift in 1991 (*t* = −25.00; *P* < 0.01), additive trends in 2001 (*t* = 32.79; *P* < 0.01) and 2002 (*t* = −38.18; *P* < 0.01), and a local trend in 2005 (*t* = 10.31; *P* < 0.01). The reduced population in 1991, by about 0.95% and in 2002 by about 2.96%, was most likely the result of negative natural population growth rate and emigration prior to the introduction of international sanctions and just after they were lifted. It also reflected the difference in the methodology used for estimating the population between the two censuses in 1991 and 2002 census years. Increased population in 2001 of 0.50% perhaps reflected the ‘pull’ effect of more popular national politics in 2000. Economic reforms and international aid that began the following year may have influenced the population decrease by about 0.30% in 2005. The ARIMA (0, 2, 0) model of GDP time-series had one local trend outlier in 1994 (*t* = 3.40; *P* < 0.01), likely a result of currency reform after hyperinflation in 1993 (which decreased GDP value by about 30.76%).

**Figure 3:**
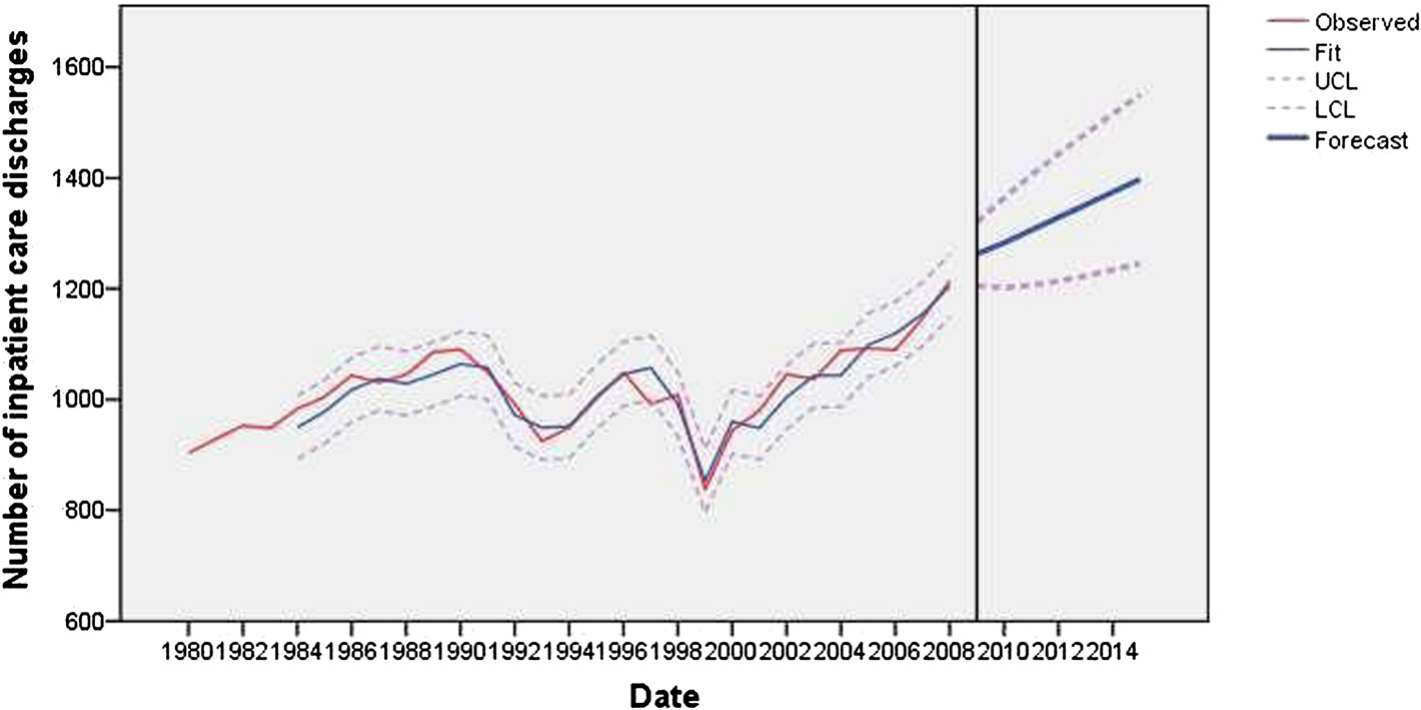
Observed and fitted number (with 95% LCL and UCL) of inpatient care discharges (x thousands) in the public healthcare sector of Serbia (1983 to 2008) and the forecast by the year 2015.

**Figure 4:**
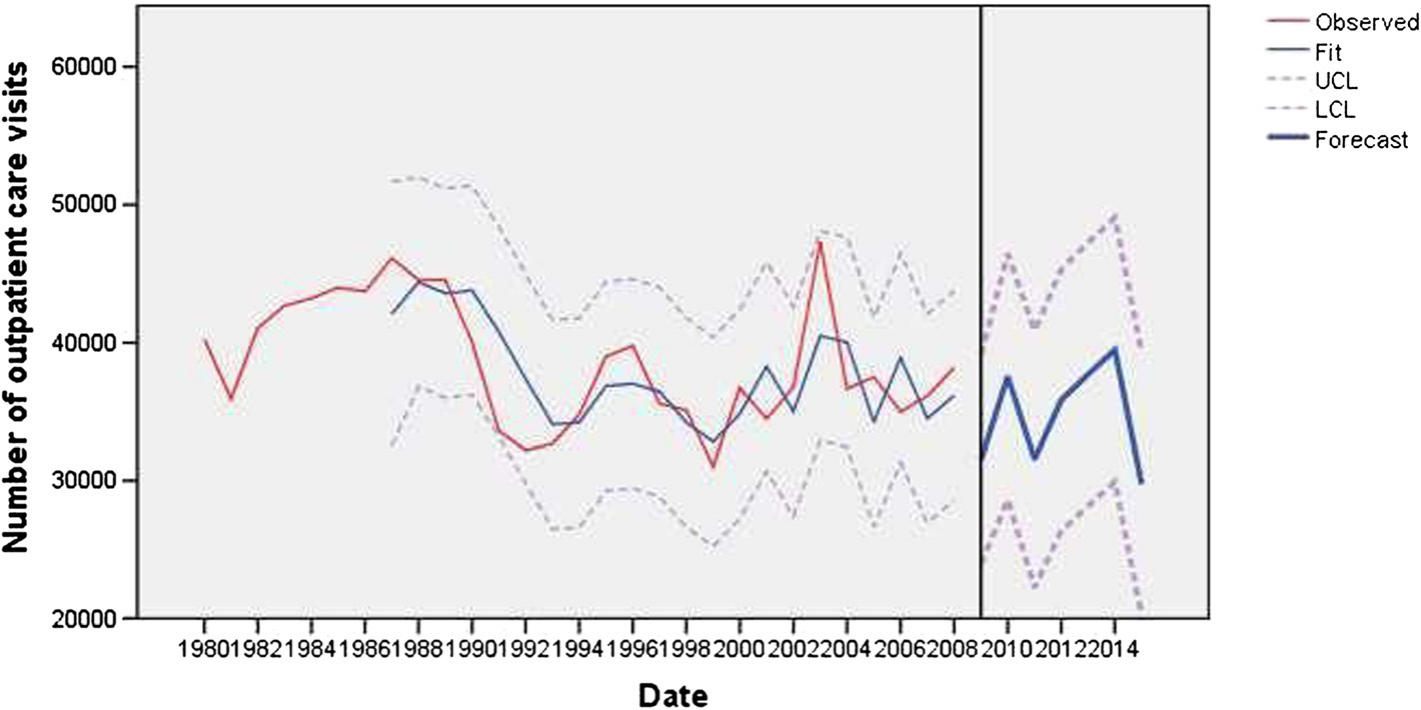
Observed and fitted number (with 95% LCL and UCL) of outpatient care visits (x thousands) in the public healthcare sector of Serbia (1983 to 2008) and the forecast by the year 2015.

The trend for inpatient care discharges had an additive outlier in 1999 by about 17.05% (*t* = −6.14; *P* < 0.01), perhaps because of reduced financial resources, GDP was a predictor and in 1999 it decreased by about 18.33%. The trend reflected the 3.30% cut back in the number of hospital beds and changed the operational plans of institutions before and during the two and a half-month long NATO bombing of Serbia (Figure 3). The model of outpatient care visits had no outliers (Figure 4).

**Figure 5:**
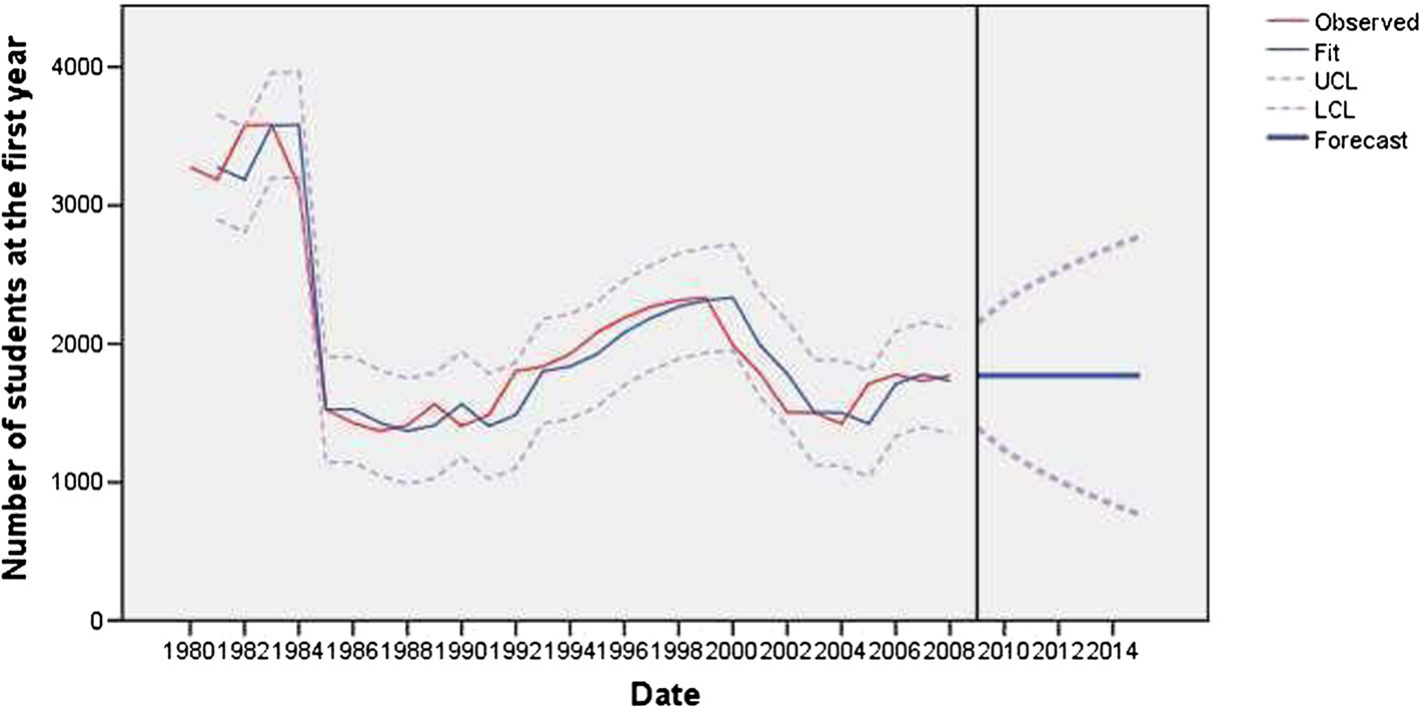
Observed and fitted number (with 95% LCL and UCL) of students enrolled in the first year of medical studies at state faculties in Serbia (1983 to 2008) and the forecast by the year 2015.

In 1985, the number of enrolled students had reverted to less than the level of 1978 (Figure 5). However, during the destabilized years of 1990 to 1994 it increased again by 30%. Shortly after political changes in the period 2000 to 2004, it decreased by 29%. In following years, it started to increase again and in 2008 there were almost 1,800 new students in the first year of state organized medical studies. These shifts make the model somewhat unstable but the long term trends can be estimated.

**Figure 6:**
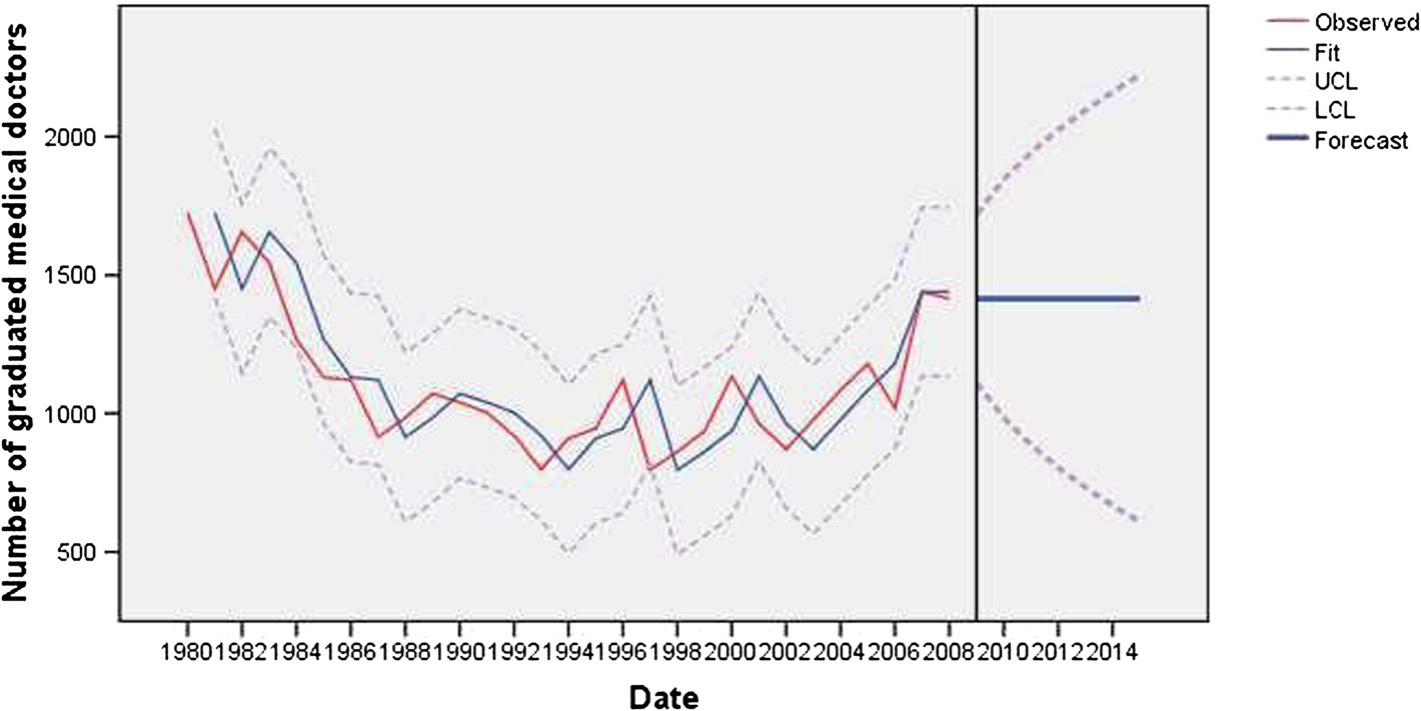
Observed and fitted number (with 95% LCL and UCL) of graduated medical doctors at state faculties in Serbia (1983 to 2008) and the forecast by the year 2015.

The number of graduates reflected intake flows. The increased number of graduated physicians in 2008 (*t* = 2.84; *P* = 0.01) was an outlier that resulted after adoption of the Bologna declaration that changed the curricula and length of medical studies (from five to six years) [25] (Figure 6).

### Forecasting the physician and nurse supply for the public healthcare sector of Serbia by 2015

According to our forecast, in 2015 there will be 1,207 more physicians (or 5.86%) and 2,459 more nurses (or 5.76%) employed in the public sector than in 2008 (Table 5). The physician and nurse rates per 100,000 of population will rise by 12.5% and by 12.8% respectively, that is, from 272 to 306 for physicians and from 562 to 634 for nurses.

**Table 5:** Forecasts with 95% confidence level and realized values of physicians’ and nurses’ supply in the public sector of Serbia through 2015

**Year**		**Physicians**				**Nurses**		
	**Forecast value**	**Lower value of 95% CL**	**Upper value of 95% CL**	**Realized value**	**Forecast value**	**Lower value of 95% CL**	**Upper value of 95% CL**	**Realized value**
2009	20983	20590	21376	20825	42062	41416	42708	42444
2010	21245	20690	21801	21054	42898	41985	43812	42722
2011	21448	20767	22128	21062	43696	42577	44815	42529
2012	21600	20815	22385		44021	42729	45313	
2013	21709	20831	22587		44677	43233	46121	
2014	21779	20818	22741		44987	43405	46570	
2015	21814	20776	22853		45111	43402	46820	

By 2015, the annual number of enrolments (1,771) will be about 20% higher than the current number of graduates at state medical faculties (1,415). The annual workforce generation ratio [13], calculated as a ratio of the number of physician graduates to the total number of physicians employed in public sector will be around 6.58% for every year in the forecast period. The increase in deployment of physicians per year is projected to be 0.7% on average. The annual differences between the new supply of graduate physicians and deployments in public healthcare sector are therefore projected to be 8,698 - a net surplus.

## Discussion

This study analyzed the relationships found in six models that described physician and nurse supply in the public healthcare system of Serbia. The models made use of over 50 years of data and estimated trends in the number of physicians and nurses required in the public health care sector of Serbia. The trends were extended to estimate the supply by 2015. This study has identified that the most significant predictors of physician and nurse staff for the last twenty-five years were GDP and population size. The relationship between changes in the economy and demand for physicians’ services was well documented in the literature [4-7]. This study revealed that the GDP is a significant predictor for the number of inpatient care discharges in Serbia, and that the supply of physicians was an incentive for healthcare service utilization in general.

Besides natural population increase, social instability and migration, other factors such as health policy (that is, changes in the health institutions’ ownership and structure, voluntary specializations, HRH rationalization and piloting new health technology), and economic changes and policy (that is, GDP, hyperinflation, currency reform and flexible retirement schemes) have affected the upward and downward slopes for physician and nurse workforce density. While changes in population have small impact on healthcare workforce, the economy had relatively greater effects (for instance, the decrease of GDP by 18% influenced downsize of the hospital bed number by 3%). Emigration also affected the public workforce density, in particular before and after the country’s break-up and during the application of international sanctions (the remaining international sanctions were lifted in September 2001). Policy changes had a major impact; for example, the HRH strategy in 1982 has reduced the intake by 51% and the staff lay-offs decreased the number of physicians by 4%. The introduction of flexible retirement as a macroeconomic intervention for staff reduction is estimated to have produced a 3.5% difference between the forecasted and registered number of health workers [9]. In our study, about 3.9% of nurses left the public sector of Serbia due to more favorable terms for retirement introduced in 1995.

Earlier studies described significant rises in the number of physicians (specialists in particular) and nurses (but not midwives) in the public sector of Serbia [23-25]. This study provided evidence that enrollment and graduation rates at state medical faculties were self-directed and caused these increases; an explicit link to the number of vacancies in public healthcare sector was not observed. The constant increase of physician and nurse density suggests that access to healthcare and to education have been traditional social values in Serbia that could resist political and economic upheavals. The prediction models showed that if current physician supply policy (enrollment and graduation at state medical faculties, and their deployment) is seen as adequate by policy makers and maintained at the same level, it will result each year in almost eight times the number of graduated physicians than the number of vacancies in the public healthcare sector. Even if almost 80% of all future graduates emigrate, or the state faculties stop the intake in the next three years, there will still be enough physicians to match the public healthcare sector requirements by 2015 and beyond. The number of unemployed physicians will likely increase. Responsive partnership between government-funded medical schools, the healthcare sector and other health stakeholders is needed [35,36]. Health experts agree that addressing population health needs should be solved by doing more than creating more health workers [35,36].

The country has spent USA$ 9 to12 billion on the education of emigrated physician specialists [37] (the lower sum corresponds to the total Serbian public debt in 2009 [38]). The real financial losses would have been much higher if the calculation covered the total estimated 10,000 Serbian health professionals working abroad [37], lost profits, replacement costs and other indirect losses. Given the actual tendency of Serbian health workers to emigrate [38], the return of investments in their education and fiscal income should be assessed.

Shifts in the macroeconomic contexts were a dominant source of forecasting failures in many studies [4,6,9,11]. To overcome this problem, in this study we included a ‘pessimistic’ scenario of GDP contraction instead of the GDP central projections since the GDP growth rate in Serbia fell from 5.5% in 2008 to −3.0% in 2009 and to 1.8% - 2.0% growth in 2010 [39]. The models in this study generated results that were fairly similar to the current situation. There was no difference between the forecasted and registered number of physicians and nurses (only 0.1% in 2011) in the public healthcare sector. This suggests that the approach can be used for projecting future workforce trends in Serbia.

### Study limitations

Models are simplifications of reality and provide a glimpse into the future based on the limitations of the models [12]. With unanticipated changes in migration of population and health workforce, the actual physician and nurse supply may also alternate. Prolonging regulations for age-related retirement in Serbia (due to an ageing workforce) may alternate the overall outflow rate from the public healthcare sector (the Ministry of Health of Serbia has estimated it at 2 to 2.5% per year [22]). Therefore, future forecasts should include the age and gender analyses of the workforce.

Having complete and valid data on the number of private practitioners is difficult in almost all countries [11,12]. In Serbia this is also a complex issue since publicly employed health workers are allowed to provide specialist, consultative and/or training services in the private sector and to act as volunteers outside the public healthcare sector. Also, according to the official methodology, only the number of full-time employees was available, while the number of consultants was unknown and likely very variable. The health professionals’ authorities regularly reported increases among licensed private health workers. The greatest proportion of private sector staff was recorded in specialty clinics, then in hospitals, women’s health care facilities and physical medicine. The climate for private business is still unstable in Serbia. Each year, a large number of new private healthcare entities enter the economy while some disappear, transform or merge (for example, in one year there were 3,000 private practices including 98 clinics and 8 primary care centers, while a year later there were 1,200 entities out of which 74 were polyclinics and 9 primary care centers) [40]. According to the assessment of the Institute of Economic and Social Research, the private sector was still poorly represented in the delivery of health care services to the Serbian population in 2009: outpatient services served 1.2% of population, and inpatient services 0.6% of population [31].

### Study implications for policy and practice

Future inter-sectoral HRH strategy and action plans should develop careful health development plans, goals and objectives. To deliver an inter-sectoral HRH strategy, the government should commission a high-level and independent body to analyze and forecast the dynamics of cross-cutting problems regarding HRH production, employment and performance. The essence of its activity should be the harmonization of HRH-related policies in all sectors with the health development plan [3,6,10].

Future research should estimate how many physicians would be employed in the private sector or abroad, and its effect in preventing future mismatches between the demand and supply of physicians in the emerging economic scenario.

Although the increasing number of unemployed health professionals suggests that there will be an available supply in the future, it also makes Serbia another source country for well- qualified health workers. The WHO Global Code of Practice on the International Recruitment of Health Personnel should be enforced in order to strategically govern the mobilization and development of a national HRH.

The number of other health professions (such as dentists, pharmacists, laboratory technicians, radiographers, physiotherapists, and so on) grew in line with the expansion of the public health sector, particularly during the extension of a guaranteed healthcare benefit basket in the mid 1970s and in the beginning of the 1980s [24,25]. Since these other professionals represent 21% of the total health workforce in the public sector, and due to their roles in teams needed to provide specialized services, their relationship with the physician and nurse staffing ratio should be explored in future research.

## Conclusions

The use of a modeling approach can help project future supply of healthcare practitioners in Serbia and help understand the balance of supply with need. The significant mismatch between the forecasted supply of physicians and available posts should be used as a pointer to decision-making on intake planning for the medical schools in Serbia. Serbia needs an inter-sector strategy for HRH development that is more coherent with healthcare objectives and more accountable in terms of professional mobility. The ARIMA-TF model may also be useful in understanding the impact of HRH governance in other countries. The relative dimension, not the specific accuracy of the continued upward trend in numbers of physicians and nurses is important for HRH stakeholders. This study may provide comparisons with other forecasts of physician and nurse supply in Serbia whilst creating future HRH policies.

## Abbreviations

ARIMA: Autoregressive Integrated Moving Average; GDP: Gross domestic product; HRH: Human resources for health; IBM: International Business Machines Corporation; LCL: Lower confidence level; NATO: North American Treaty Organization; SPSS: Statistical Package for the Social Sciences; TF: Transfer function; UCL: Upper confidence level; USA $: United States of America Dollars; WHO: World Health Organization

## Competing interest

The authors declare that they have no competing interests.

## Authors’ contributions

MSM, VV and JM conceived and designed the study and have made substantial contributions to analysis and interpretation of data. MSM and VV collected data and drafted the manuscript. All authors have given the manuscript their final approval.
